# Rural Hygiene

**Published:** 1878-08

**Authors:** 


### Rural Hygiene.

The present season naturally suggests the feasibility of a temporary absence from the city and its possible advantages. Although the practice of ruralizing is becoming more and more extensive, the evils which have become associated with it have been proportionally magnified. In consequence of the demand for country board, a large number of farmers have thrown open their doors for customers, and view with the country hotel-keeper in the general unsanitary condition of their premises. Even in the so-called model farmhouses, the first-class country hotels, and in the larger establishments of the more fashionable resorts, we often find the foul and reeking privy vaults in close proximity to a well; the rooms, if not small and close, at least unventilated; while the dietary is stamped with the most objectionable features of boarding-house management. Under the circumstances, except for the outdoor life which the season invites, many of the guests would suffer from serious attacks of illness. The religious camp-grounds, in many instances, need much more sanitary supervision to make their continued occupation from year to year free from danger.

All these are certainly drawbacks; but there is no doubt that the benefits of ruralizing are sufficient to warrant every hard-worked, nervous, overstrained Metropolitan in being temporarily absent from his home—free for a time from business cares, and free to enjoy a change of scene and condition, which, at this season of the year particularly, he so much demands.—*Med. Record.*

